# Investigation of the Quasi-Static Penetration Resistance Behaviour of Carbon/Aramid Fibre-Reinforced PP Laminate

**DOI:** 10.3390/ma14040709

**Published:** 2021-02-03

**Authors:** Joanna Pach, Ewa Kuterek

**Affiliations:** Department of Lightweight Elements Engineering, Foundry, and Automation, Faculty of Mechanical Engineering, Wrocław University of Science and Technology, Wyb. Wyspiańskiego 27, 50-370 Wrocław, Poland; ewa.kuterek@gmail.com

**Keywords:** hybrid composites, laminates, polymer–matrix composites, quasistatic penetration test

## Abstract

This work presents the experimental results of a quasi-static attempt at the penetration of hybrid and non-hybrid laminates reinforced with aramid and carbon fibres on a thermoplastic polypropylene matrix. The hybrid laminates were prepared in two fibre combinations: carbon–aramid–carbon (CAC), in which the carbon fibres comprised the outer (lining) layers, and aramid–carbon–aramid (ACA) with carbon fibres in their intermediate layers. A quasistatic penetration attempt was performed for two coefficients: SPR—support span to punch diameter ratio, (SPR = 2 and 5). The SPR = Ds/Dp was calculated as the ratio of the support (Ds) to the punch diameter (Dp). A punch with a rounded 9-mm diameter tip was used to penetrate the material. Percentage changes of penetration energy (%E) and of maximum load (%P) compared to a non-hybrid laminate with carbon fibres were calculated in order to estimate the impact of hybridisation on the properties of laminates. The maximum load recorded during a quasi-static penetration test was used to calculate the PSS (punch shear strength) of the laminates. The damage was observed after the penetration test. It was observed that both the order of layers of laminate reinforcement as well as the SPR coefficient used in the test influenced the obtained results and the laminate damage mechanism.

## 1. Introduction

Hybrid laminates are a special group of polymer composites. Due to the combination of at least two different reinforcement types, laminates may be characterised by high resistance and rigidity with a relatively low cost of manufacture.

Laminate hybridisation involves the use of various fibres as reinforcement. This allows composites to be obtained that combine the properties of the reinforcing fibres used. Hybridisation used in laminates may be an effective method of improving the ability to absorb energy. This may be important in the case of laminates used in the automobile industry and in ballistic laminates. 

Attempts at the evaluation of the hybridisation effect are documented in the literature; the effect may be determined in a penetration test on the basis of the value of energy absorbed by the hybrid laminate compared to the average value of energy absorbed by composites including a single reinforcement type. Calculations of the hybridisation effect related to the rule of mixture have been presented, i.e., in the work by Bulut et al. [1].

In the experiment performed by the authors [1], laminates with reinforcement made of carbon, aramid, and glass fibres were used. Laminates and hybrid laminates on an epoxy matrix were prepared, with different configurations of the reinforcement layers used. This work included quasi-static penetration tests (QSPT), and the hybridisation effect was calculated. The most profound, positive hybridisation effect was recorded for laminates in which carbon fibres were present in the outer layers with aramid fibres present between them. On the other hand, the highest negative hybridisation effect was recorded for a laminate in which carbon fibres were present in the outer layers while glass fibres were present in the inner layer. It was concluded that fibres present in the outer layer have a significant influence on energy dissipation and on the mechanism of structural damage. 

Similar studies on hybrid laminates consist of aramid and kenaf fibres were also described [2]. The authors observed that in the case of laminates with aramid fibres in the outer layers and kenaf fibres in the inner layers of the composite, the values of absorbed energy were higher than for other tested samples. The values of energy absorbed during penetration of hybrid laminates were higher than those for non-hybrid laminates with only kenaf or aramid fibres. This work also calculated the hybridisation effect. Contrary to the aforementioned work by Bulut et al. [1], a different method was used to calculate the hybridisation effect. The influence of hybridisation of the aramid laminate using natural fibres was calculated separately for the absorbed energy and the maximum transferred load. These values were calculated in relation to a sample of a non-hybrid aramid–epoxide composite.

The influence of hybridisation on mechanical properties of composites was also described in other works [3,4]. Bandaru et al. studied the impact of hybridisation on ballistic resistance of composites. On the other hand, the work by Pérez-Fonseca et al. studied the impact of hybridisation on the mechanical properties of composites with natural fibres using high-density polyethylene matrix. 

Composites based on a polyethylene matrix were also described in the work by Erkendirci et al. [5]. Carbon fibre/polyethylene (PEHD) laminates with two different thicknesses were produced and subjected to a quasi-static penetration test. The tests were performed at various SPR coefficients with values in the range between 1.16 and 6.67. It was found that the higher the SPR coefficient used, the higher the value of total absorbed energy. These results coincide with conclusions presented elsewhere [1], where it was observed that the penetration force, tangential strain, and maximum value of absorbed energy increased with the increasing SPR coefficient.

Quasi-static penetration tests were used to study progressive damage to carbon–epoxy laminates [6]. The SPR coefficient was constant here and equal to 2, but different loads were used with individual samples. A damage analysis was performed using a microscope and a computer tomograph. It was found that laminate load results in a change of local shearing into a full bending. The ultimate damage of laminate was the result of large delamination and the influence of shearing and stretching loads. 

The results of a quasi-static test may be used to study the damage mechanism and to predict the dynamic behaviour of the material. Studies related to this topic were performed in works [7,8]. It was observed that the ratio of impact force to the offset was identical in the quasi-static test and in a low-velocity impact test, namely a test performed in the range 1–10 m/s. Very small dynamic loads, which may be neglected during the analysis, occur during the low-velocity impact test. 

Gama and Gillespie [9] evaluated the laminate damage mechanism using a quasi-static test and a ballistic test. A QS-PST experimental methodology was developed to model different phases of ballistic penetration and was used in evaluating the quasi-static energy-absorbing behaviour of composite materials as a function of thickness. The work by these authors involved the damage characterisation of a composite reinforced with glass fibres, both in the quasi-static test and in the low-velocity penetration test, as well as in the ballistic test. The quasi-static tests were used as the basis to develop a ballistic penetration model, and it was calculated that the total energy absorbed during a QSPT comprises 81% of the total absorbed energy measured during ballistic experiments. This information cannot be extrapolated to thermoplastic behaviour. 

The work by A. Wagih et al. [10] included a quasi-static test intended to explain the damage sequence present during low-velocity penetration. The test was performed using laminates reinforced with carbon fibres, with an epoxy matrix. Four damage stages in the studied material samples were defined after a microstructural analysis using an electron microscope and after a non-destructive ultrasound test. The elastic strain of the sample without damage was observed during the first stage. The second stage was characterised by a sudden drop of load related to the initial cracking of the matrix. Matrix cracks were located at the points of the main strain focus. Delamination at the ends of matrix cracks was observed in the third stage. The fourth stage included an energy drop related to fibre damage. It was found that most of the energy is dispersed during this stage, and the total destruction of the sample occurs. 

To determine the properties and the dynamic reaction of the material, a quasi-static test, low-velocity penetration, and a ballistic test were compared [11]. In addition to the QSPT test, ballistic testing of hybrid laminates was also described in some works [2,3,12].

Currently, laminates and hybrid laminates are being tested by various researchers, both in QSTP tests and in ballistic tests. Determining a correlation between the behaviour of a laminate in the QSTP test with low-velocity penetration and in the ballistic test remains a challenge and a current research topic for numerous researchers. The obtained study results may be extremely helpful when designing and testing laminates. Due to the limitations of experimental techniques, above all, the high cost of ballistic tests, it is reasonable to conduct quasi-static puncture tests, which in the future may be useful in the development of damage models occurring at higher penetration velocities.

The aim of the authors is to develop lightweight shields that protect against the effects of low-speed impact or laminates that are the back layers of ballistic composites. Aramid fibres are a better choice than carbon fibres in composites used, e.g., for puncture protection. However, the price of aramid fibres is high. In some applications, aramid can be replaced with carbon fibres, while maintaining a beneficial price/properties ratio. In order for such a hybrid composite to be light, polypropylene was chosen as the matrix, among others, due to its low density.

The aim of this work was to analyse the damage and to determine the impact of hybridisation for hybrid laminates with polypropylene matrix, with two reinforcement types: aramid fibres and carbon fibres. QSPT and calculations of the hybridisation effect for SPR coefficient values 2 and 5 were planned. Similar experiments were performed by the aforementioned authors [1,2] for laminates on an epoxy resin matrix, but the results cannot be extrapolated to the thermoplastic behaviour.

The authors of this work, however, selected polypropylene as the thermoplastic matrix for the tested laminates. The selection of polypropylene as the laminate matrix was inspired by a work of Carillo et al. [13], in which a positive impact energy absorption effect was observed when polypropylene was introduced between aramid textile layers. The use of polypropylene as a matrix in laminates with aramid fibres may offer an interesting alternative to laminates with cured resin matrices, e.g., matrices made of epoxy resins [14,15]. Polypropylene may be used as a matrix in composites, both with aramid fibres and with glass, natural, and metal fibres [16,17,18]. Work [19] presents an interesting comparative analysis of laminates with glass fibres on polypropylene and on an epoxy resin matrix. Literature analysis shows, however, that the effects of hybridisation of laminates based on polypropylene matrix with the reinforcement type proposed in this work have not been compared yet.

The hybridization effect using polypropylene as a matrix is not obvious due to the different properties of polypropylene compared to epoxy resin-based laminates, widely described in the literature. Therefore, the research and evaluation of the hybridization effect of laminates on a polypropylene matrix confirm the novelty aspect of this work. The authors of this study described the puncture of polypropylene and polyethylene matrix laminates, which were ballistic tested and analysed in detail by computed tomography [20,21]. The study presents the X-ray computed tomography (XCT) analysis for structure assessment of ballistic panels and its impact behaviour, further compared to the results of computer simulations conducted using the numerical analysis [21]. 

Previous research, however, focused on non-hybrid laminates with only aramid reinforcement. As the previous test results showed, the choice of the polymer matrix was very important for the achieved results, because the polypropylene matrix laminates stopped the projectile. This result was not achieved with laminates based on a high-density polyethylene.

Taking into account the results of previous work, it was decided to analyse hybrid laminates on polymer matrix other than epoxy resin. This paper presents the results of a quasi-static puncture test for laminates on a polypropylene matrix. In addition, quasi-static puncture tests were also carried out for laminates based on a polyurethane-polyurea matrix [22].

## 2. Material and Methods 

### 2.1. Sample Preparation

Laminates reinforced with aramid textiles (AAA), carbon textiles (CCC), and hybrid laminates using both these reinforcement types (CAC, carbon–aramid–carbon and ACA, aramid–carbon–aramid) were prepared for the tests, i.e., aramid textile with a plain weave and with areal density of 173 g/m^2^ (Twaron, distributed by Havel Composites, Cieszyn, Poland) and carbon textile (Kordcarbon) with a plain weave and with areal density of 200 g/m^2^. Polypropylene granulate (PP) HP 548R (Basell Orlen Polyolefins, Płock, Poland) was used as the polymer matrix. The mechanical properties of the textiles and of the polymer matrix are summarised in Table 1.

**Figure 1:**
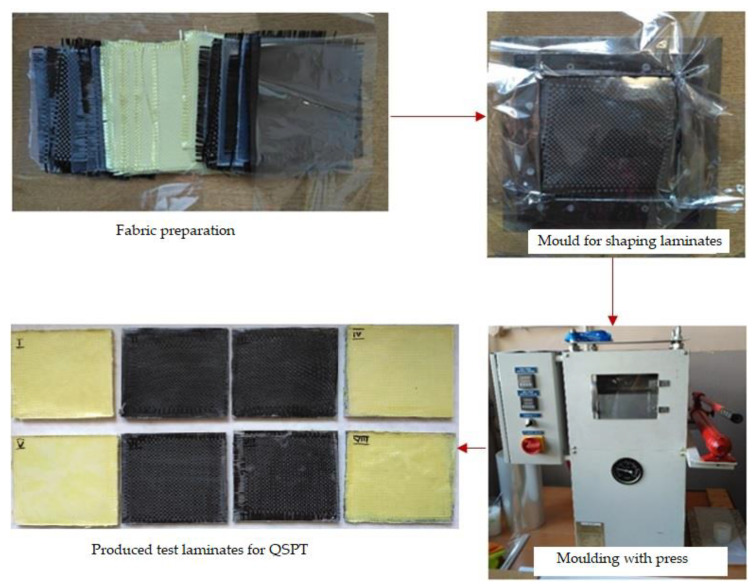
The process of producing laminates under a hydraulic press.

The laminates were formed in two stages. The first stage was the formation of the film from polymer granulate which initially was plasticised under a press at a temperature of 200 °C for 2 min without load, and then for 2 min under a pressure of 2 MPa. As a result of pressing, polymer films were obtained. The laminates were produced by the alternate pressing of 15 layers of polypropylene film and 14 layers of fabric arranged in a metal mould. The laminates were moulded at a temperature of 200 °C. They were pressed for 2 min with no load and for 3 min under a pressure of 5 MPa. Figure 1 presents the laminate preparation process.

**Figure 2:**
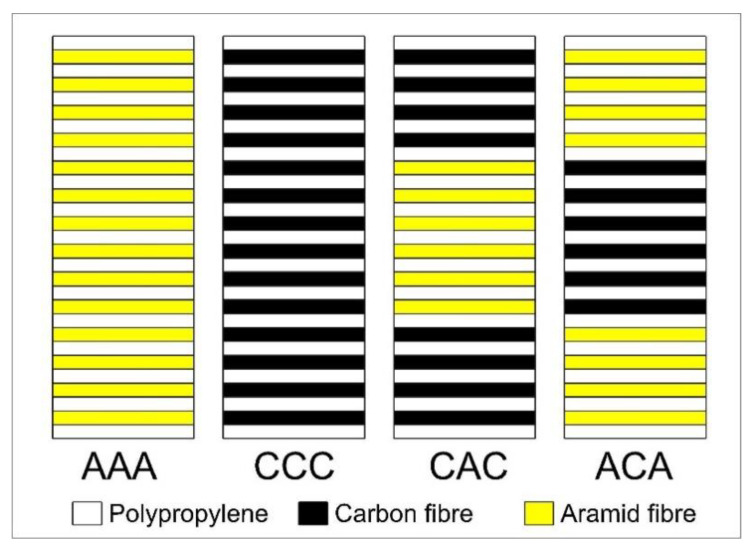
Reinforcement layer configuration in laminates.

The stacking sequences (Figure 2) and fibre fractions (% mas.) are given in Table 2. A series of non-hybrid laminates, including reinforcement in the form of aramid or carbon textiles only, was prepared, as well as hybrid laminates, in which aramid fibres were present in the outer or inner layers of the laminates. 

### 2.2. Quasi-Static Laminate Penetration Test

**Figure 3:**
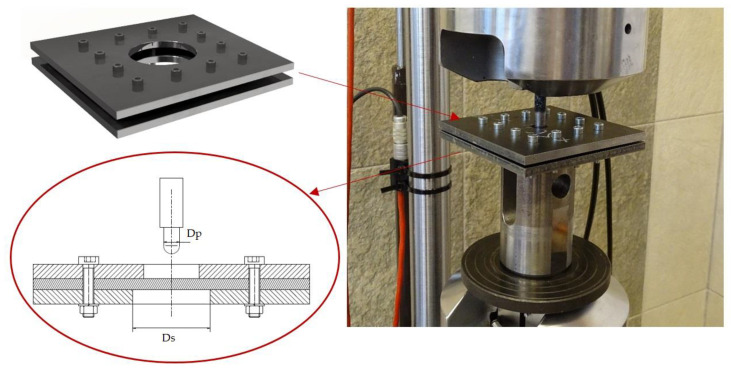
Penetration test equipment. Dp—punch diameter, Ds—support diameter.

The quasi-static test was performed using an MTS 810 resistance testing machine (Wrocław, Poland). A punch, with a diameter of 9 mm, was installed in the top jaw of the resistance testing machine, representing the shape of a Parabellum round. The punch was made of steel with a tempered surface. The penetration test equipment is schematically presented in Figure 3.

The test setup included two panels: a square cover and a support plate 150 × 150 mm^2^, 8 mm thick. Holes were cut out in the middle of both panels. Then, 100 × 100 mm^2^ laminate samples were attached between both panels of the test setup using 12 screws. The displacement rate of the punch was 1.25 mm/s. The puncture test was performed at room temperature, according to ASTM D732 standards [23]. 

The penetration test was performed at two values of SPR: 2 and 5. SPR = Ds/Dp, where Ds is the diameter of the hole in the support panel and Dp is the punch diameter. Four laminate types were tested for each of the SPR values.

The test was intended to analyse the damage stages as a function of the SPR coefficient and to determine the impact of hybridisation and of the reinforcement layer sequence on the value of absorbed energy and of the PSS. 

The PSS was calculated according to Formula (1) [1].

PSS = Pmax/(πDp Hc)(1)

where: Pmax—maximum force, Dp—diameter of the punch, Hc—thickness of the laminate.

The total energy (Ea) absorbed by the laminate during penetration may be calculated as the surface area under the force–displacement curve [1]. (Ea) is the sum of the energy absorbed by the laminate in three damage areas, i.e., elastic energy, energy in the damage area, and friction energy. The total absorbed energy (Ea) was determined using the MatLab suite (R2017bversion), using the trapezium integration method.

## 3. Results

### 3.1. Quasi-Static Laminate Penetration Test

**Figure 4:**
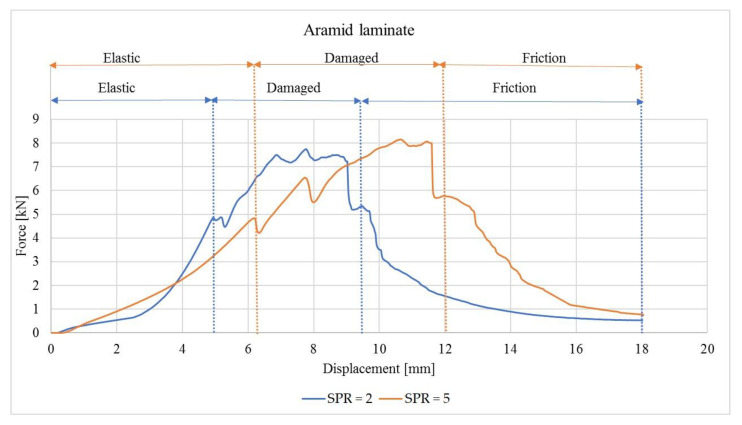
Force as a function of displacement for laminates on a polypropylene matrix with aramid fibres, obtained for support span to punch diameter ratio (SPR) = 2 and SPR = 5.

Experimental tests were performed for two coefficients: SPR = 2 and SPR = 5. Figure 4 presents the force–displacement curve for laminates reinforced with aramid fibres.

The force–displacement curve recorded during the laminate penetration test has characteristic areas marked, corresponding to damage stages: the elastic part, the damaged part, and the friction part. During the first phase, with the load increasing linearly, elastic deformations are observed. The polymer matrix breaks and delamination occurs during the next stage. During the damage phase, the curve shows numerous peaks corresponding to fibre shearing and breakage. A plug is formed at the point where strain accumulates. The last part of the force–displacement curve is related to shearing between the laminate and the punch. 

Yahaya et al. [2] performed a quasi-static kenaf–aramid laminate penetration test on an epoxy matrix and also distinguished three areas on the force–displacement curve: elastic, damage, and friction. On the other hand, Wagih et al. [10] tested laminates with carbon fibre reinforcement and epoxy resin. The authors distinguished four stages: I—elastic, II—matrix breakage, occurring after the peak related to decreasing strain, III—delamination propagation, and IV—fibre breakage. 

In this work, three stages of laminate destruction were distinguished along each of the force–displacement curves. The load increases up to a critical value during the quasi-static penetration test, and then it decreases rapidly. Fibre damage and laminate perforation occur, and plugging begins during the next stage, followed by shearing. 

**Figure 5:**
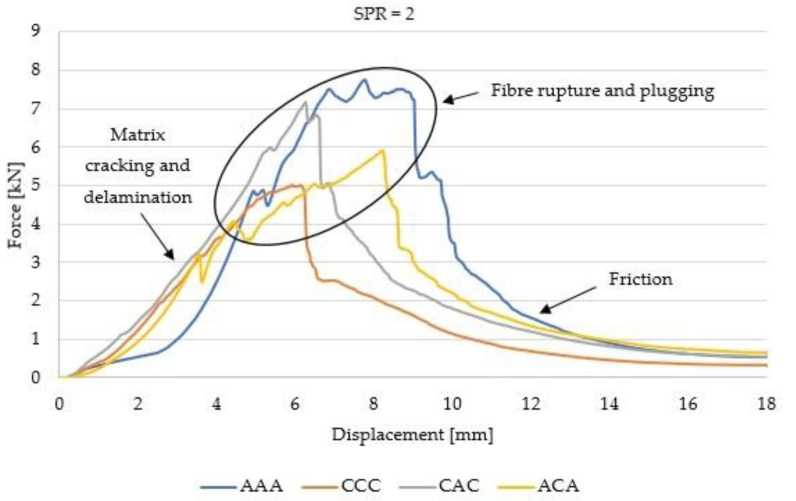
Force–displacement curves recorded during the quasi-static penetration test (QSPT) for SPR = 2.

**Figure 6:**
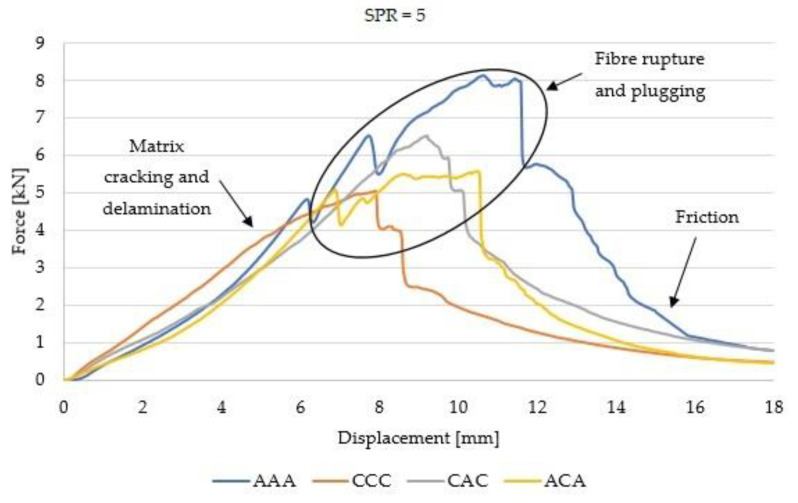
Force–displacement curves recorded during the QSPT for SPR = 5.

Figure 5 and Figure 6 present the force–displacement curves for hybrid and non-hybrid laminates. 

The force–displacement curves recorded for SPR = 2 and 5 are different. In the case of SPR = 5, the damage area is larger and the peak corresponding to Pmax is shifted to the right compared to SPR = 2. This observation is confirmed for all laminates compared in this work and is in agreement with the results recorded by Bulut et al. [1]. The size of the damage area is influenced by the support diameter (Ds) and by the laminate area subjected to strain related to penetration. The quasi-static damage mechanisms are a function of support span: small represents shear dominated damage, and a large represents bending dominated damage [9].

Other differences observed on the curves include the fact that in the case of non-hybrid aramid laminates (AAA), the damaged area is larger than in the case of carbon fibres (CCC). Differences in the force–displacement curves were also observed for hybrid laminates. In the case of hybrid laminates with aramid fibres in the outer layers (ACA), the curve is less steep in the destruction area, while in the case of CAC laminates, higher values of peak loads were observed. These observations are valid regardless of the SPR value. These differences may be explained by the different properties of the reinforcing fibres, and more generally, by the overall rigidity and ductility of aramid and carbon fibres. Aramid fibres are more ductile and more susceptible to deformations in the penetration test. 

**Figure 7:**
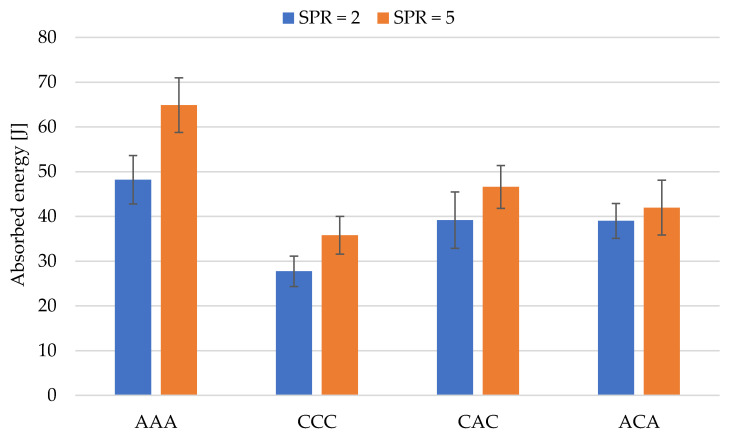
Comparison of absorbed energy (Ea) values for hybrid and non-hybrid laminates at SPR = 2 and 5.

**Figure 8:**
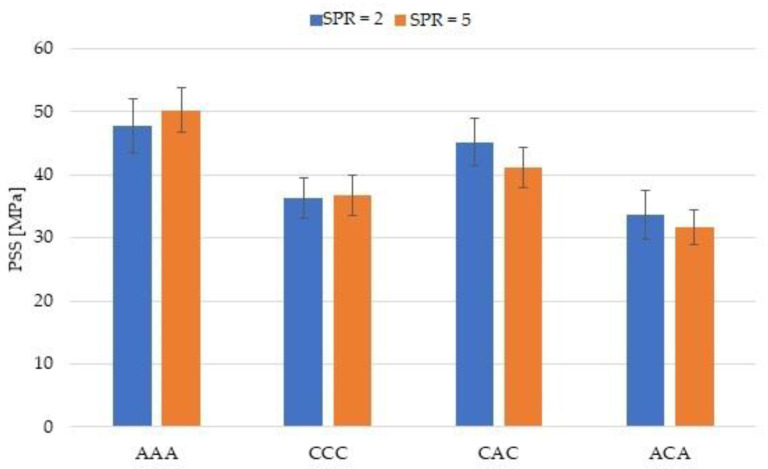
Punch shear strength (PSS) comparison for hybrid and non-hybrid laminates at SPR = 2 and 5.

The results of the experiment performed in the QSPT test are presented in Table 3. Additionally, Figure 7 and Figure 8 present the calculation results for absorbed energy (Ea) and punch shear strength (PSS).

Specific energy absorption (SEA) is the energy absorbed per the mass of the specimen. SEA was calculated from the equation: (SEA = TEA/mass) [24]. The values of total absorbed energy (Ea), specific energy absorption (SEA) and of maximum force (Pmax) are clearly higher for aramid laminates, both at SPR = 2 and 5. The highest value of absorbed energy (Ea), specific energy absorption (SEA) and of maximum force (Pmax) was achieved for the aramid laminate at SPR = 5. The value (Ea) is nearly 45% higher than the energy for carbon laminate at the same SPR coefficient. 

The values of absorbed energy for SPR = 5 are higher than the values achieved at SPR = 2, both for non-hybrid and hybrid laminates. The higher values of total absorbed energy for SPR = 5 are related to the greater diameter of the hole provided in the support plate. The surface area on which the punch interacts with the laminate is then larger. 

Figure 7 and Figure 8 compare Ea and PSS results for hybrid and non-hybrid laminates, for SPR 2 and 5. 

In the case of hybrid laminates, the (Ea) and (PSS) values were generally higher for laminates in which carbon fibres were present in the outer layers of the laminate (CAC). The conclusions resulting from the calculations of specific energy absorption for different laminates are completely consistent with the results of total energy absorption presented in Figure 7. It results from similar masses of the analysed laminates. The presentation of the SEA results was limited to presenting the results in Table 3. The comparison of Ea values for SPR = 2 is an exception, with values of both hybrid laminates being comparable. In the case of SPR = 2, the PSS value for the CAC laminate is by nearly 25% higher compared to the ACA laminate and 8.51% lower than the aramid laminate. Comparison of Ea and PSS values thus leads to the conclusion that the hybrid laminate in which carbon fibres comprise the outer layers (CAC) is more effective. Similar results were obtained in a quasi-static penetration test (QSPT) for aramid–carbon hybrid laminates based on a polyurea–polyurethane matrix [22]. Higher Pmax, Ea, and PSS values were recorded in this work for laminate in which carbon fibres comprised the outer layers. 

The differences in the behaviour of the laminate during penetration are a result of different flexural stiffness of the fibres. The use of aramid fibres as the inner layers of the laminate exhibited maximum peak loads. The explanation behind the more favourable configuration of CAC fibres in hybrid laminates may be found in the bending theory. 

The compressive strength of the first laminate layers is important. Carbon fibres show a higher Young’s modulus than aramid fibres, therefore their position in the first layers of the laminate seems to be more favourable in the laminate. In the next phase of punching, the reinforcement layers are sheared and stretched. After crossing the neutral layer of the laminate, the fibres are stretched. High strength and elongation are desirable characteristics of the fibres used in puncture-resistant laminates. Aramid fibres are more ductile than carbon fibres, but the elongation of aramid fibres can result in additional failure mechanisms such as delamination.

### 3.2. Macroscopic Analysis of Laminate Damage after the Quasi-Static Penetration Test

Photographs of the front and the rear surfaces of laminates were taken after the quasi-static penetration test. Laminate destruction related to fibre shearing and breakage, matrix cracking, and delamination was observed. By comparing laminate damage for SPR = 2 and SPR = 5, the formation of a characteristic cross-like pattern on the rear side of laminates penetrated at SPR = 5 was observed. The results of the observed laminate damage are summarised in Table 4. The samples were cut transversely at the puncture location in order to better understand the mechanism behind damage occurring during the QSPT. The cut was performed using a water jet. Photographs of laminate cross-sections were taken using a 3× zoom and are also presented in Table 4.

Comparison of laminate damage at SPR = 2 and SPR = 5 indicates that the support length and the exposed laminate surface influence the destruction mechanism. A larger surface area of damage was observed for SPR = 5. The visible cross-like pattern on the rear surface of laminates indicates a damage mechanism related to the stretching of primary fibres. Fibre stretching is related to laminate bending here. In the case of laminates penetrated at SPR = 2, fibre damage was caused mainly by shearing and compressing strain. 

In the photographs of the laminate cross-section at the penetration point, fibre shearing may be observed in the top part of the laminate in the initial stage, followed by stretching of intermediate and inner layers of reinforcing fibres. Fibre destruction in the rear (inner) part of the laminate is caused by stretching forces present during bending. Damage recorded during the test included the following: polymer matrix breakage, fibre shearing, breaking and bending. In the case of CAC laminates in which the outer layers were made of carbon fibres followed by aramid fibres, a raised layer of the carbon fibres is visible at the interface between the layers of carbon and aramid fibres, accompanied by the formation of a void at the contact point with the layer of aramid fibres. This is caused by the different mechanical properties of the fibres. This effect is not as profound in the case of the fibre layout in the ACA configuration, which could mean that inclusion of carbon fibres in the initial layers, followed by aramid fibres, may result in an additional destruction mechanism, such as delamination.

**Figure 9:**
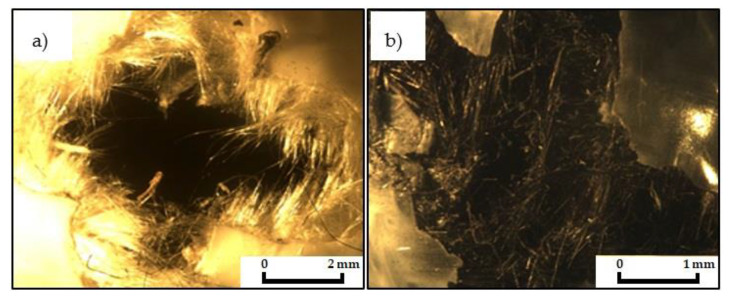
Photographs of laminate puncture locations for (**a**) aramid AAA, (**b**) carbon CCC.

The laminate puncture location was observed under a Stemi 2000-C biological microscope (Wrocław, Poland, Country), at 6.5× magnification. Figure 9 presents the puncture locations for two selected laminates.

Thermoplastic matrices are considered to have lower impregnation rates compared to epoxy resins Figure 9a,b show that not all bundles of carbon and aramid fibres are wetted by the PP matrix. Similar observations were described by Erkendirci [5] for laminates with carbon fibres on a PEHD matrix. 

The differences with the use of a thermoplastic matrix compared to a thermoset matrix are very significant, which affects the results of impact tests. The thermoplastic-based laminates showed less damage area and less damage propagation, in comparison with thermoset-based laminates. This confined damage area under single and recurring strike impact in the thermoplastic composites is attributed to higher interlaminar fracture toughness, crack resistance, and matrix ductility, which suppresses damage propagation [25].

### 3.3. The Hybridisation (Hybrid) Effect

Studies related to the influence of hybridisation on the properties of a composite have already been described [25]. Problems related to the definition of the hybrid effect were described in detail in the work by Swolfs et al. [25]. Some authors calculate the hybridisation effect as a positive or a negative deviation of mechanical properties from the rule of mixture [1]. Application of the rule of mixture in such calculations, however, is not simple. This rule is not always linear for all properties. Additionally, application of the rule of mixture requires a specific composition parameter for the studied composites. Examples of such parameters include relative volumetric shares of fibres with LE—long elongation and of fibres with HE—high elongation. Experimental determination of such parameters, however, is not always easy [25].

In this work, the hybrid effect was calculated according to Formulas (2) and (3), described already in other works [2,26] and modified for the studied laminates. The hybrid effect achieved: percentage changes of penetration energy (%E), of the maximum load (%P) and (%PSS) (Formula (4)) was calculated as follows:

(2) $$\%E=\left(E_{h}-E_{C}\right)/E_{C}\times 100\%$$

(3) $$\%P=\left(P_{h}-P_{C}\right)/P_{C}\times 100\%$$

(4) $$\%PSS=\left(PSS_{h}-PSS_{C}\right)/PSS_{C}\times 100\%$$

where

*E_h_*, *E_C_*—Energy absorbed by the laminates: hybrid and carbon laminates;
*P_h_*, *P_C_*—Maximum force (Pmax) for the hybrid and the carbon laminate,
*PSS_h_*, *PSS_C_*—Punch shear strength (PSS) for the hybrid and the carbon laminate.

**Figure 10:**
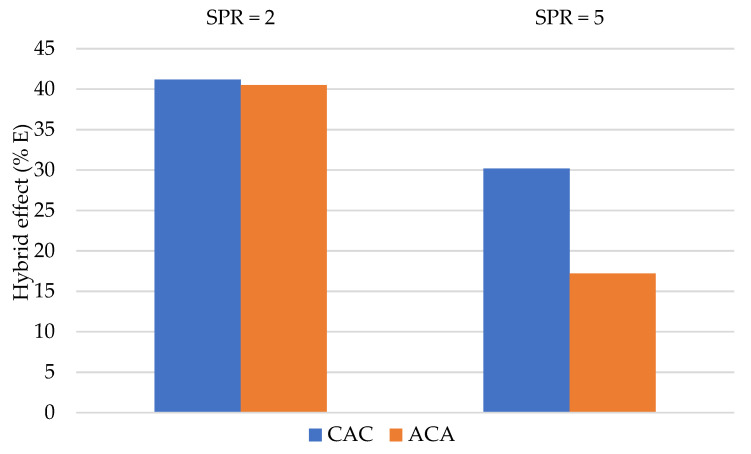
The percentage changes in absorbing total energy due to hybridisation.

**Figure 11:**
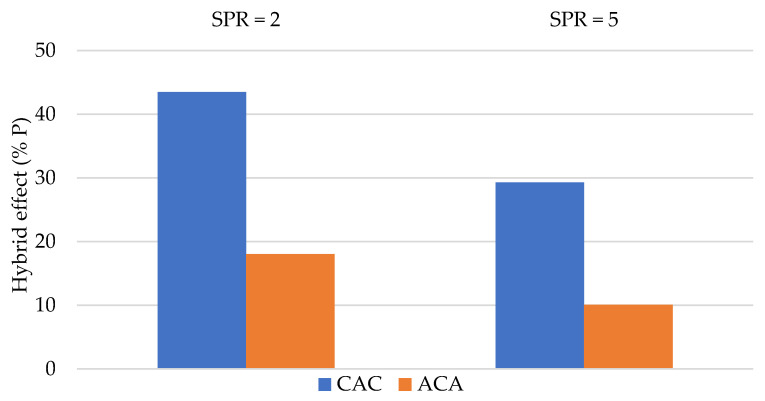
The percentage changes in maximum carried load (Pmax) due to hybridisation.

Figure 10 and Figure 11 present changes of the absorbed energy Ea and Pmax as a result of laminate hybridisation.

The biggest positive effect of hybridisation was observed for CAC laminates in which carbon fibres formed the outer layer. This observation was repeated in the case of both SPR = 2 and SPR = 5, during calculation of the hybridisation effect on the energy absorbed and on Pmax. Similar results were presented in a different work of author, which studied hybrid aramid–carbon laminates on a polyurethane–polyurea matrix [22]. 

The obtained results indicate that placement of carbon fibres on the impact side is beneficial. This may be explained by differences in the damage mechanisms between carbon and aramid fibres. 

The first layers of the laminate are compressed during penetration in the quasi-static test; thus, the compression strength of the outer laminate layers is quite important. Compared to aramid fibres, carbon fibres are characterised by a higher Young’s modulus; thus, their presence in the outer laminate layers seems to be more beneficial. 

During the next stage of punch penetration into the laminate, the fibre layers are sheared and stretched. During the last stage, once the neutral layer is penetrated, fibre layers become stretched. Use of fibres with high resistance and elongation seems to be justified here. These are properties characteristic for aramid fibres. Aramid fibres (HE) are more ductile than carbon fibres (LE). Aramid fibre elongation may cause additional damage mechanisms in the laminate, such as layer separation. Similar conclusions were reached by Park and Jang [27], who observed higher strength to impact in carbon–aramid laminates if carbon fibres were present on the impact side. The authors explain that this layout allowed the aramid fibres present on the stretched side of the sample to absorb more energy.

Bulut et al. [1] observed the highest positive hybridisation effect when aramid fibres comprised the inner layer of the laminate while carbon fibres formed the outer layers. The authors calculated the hybrid effect differently (using the rule of mixture) than the authors of this work; however, the related conclusions are similar. 

**Figure 12:**
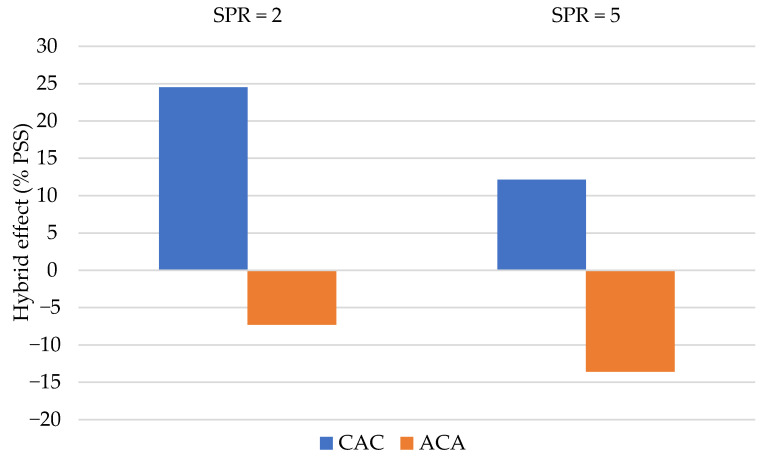
The percentage changes PSS due to hybridisation.

Finally, calculations of the influence of hybridization on the PPS value were performed. In this work, this result should be taken into account because the laminates differed in thickness. Figure 12 shows the results of these calculations. The hybridization effect (% PSS) for ACA laminates is negative and this is due to the difference in thickness of the tested laminates, which is taken into account in the PSS calculations.

In summary, the layout of fibre layers in a hybrid laminate is quite significant because it influences rigidity and flexural strength, as well as laminate damage mechanisms. 

## 4. Conclusions

This work analysed the influence of hybridisation on the ability of composite materials to absorb energy. Laminates and hybrid laminates with different fibre configurations were prepared. Aramid and carbon textile was used as a reinforcement, and a polypropylene matrix was used. The materials were subjected to a quasi-static test at the SPR coefficient values of 2 and 5. Damage after penetration was analysed, and the puncture strength and total absorbed energy were calculated. The following conclusions were formulated:

(1) As a result of macro- and microscopic evaluation of laminate destruction areas after the quasi-static tests, damage such as delamination, fibre shearing, matrix cracking, and plug formation after penetration was observed. Differences in destruction and damage of carbon and aramid fibres were observed, depending on their location within the laminate.
(2) Penetration in aramid–carbon hybrid composites includes layers of fibres of different stiffness and coefficient of friction. Thus, the sequence in hybrid laminates plays an important role in penetrating the laminates.
(3) The first reinforcement layers were destroyed as a result of shearing, while fibre damage in the inner layers of the laminate was caused by stretching forces.
(4) The highest value of absorbed energy (Ea), specific energy absorption (SEA) and of the maximum force (Pmax) was recorded for non-hybrid aramid laminates, regardless of the SPR coefficient.
(5) The layout order of fibres within the laminate influenced its effectiveness in the penetration test and the destruction mechanism. It was observed that the presence of carbon fibres in the first layers of the laminate, followed by aramid fibres, resulted in an additional destruction mechanism in the form of delamination at the junction between the two reinforcement types.
(6) In the case of hybrid laminates with the CAC configuration, compared to ACA, a higher value of total energy (Ea), specific energy absorption (SEA) and the hybrid effect calculated in relation to the amount of energy absorbed and the Pmax value were achieved. Similar results were obtained, regardless of the SPR coefficient.
(7) The SPR coefficient influenced the laminate destruction mechanism in the QSPT test. It was observed that at the higher SPR = 5, the fibre damage mechanism was related more to the bending of the laminate and the stretching of the fibres, while at SPR = 2, fibre damage was caused mainly by shearing and compressing strain.

## Figures and Tables

**Table 1:** Properties of laminate ingredients.

Fibres/Polymer Matrix	Density (g/cm^3^)	Tensile Strength (MPa)	Young’s Modulus (GPa)
Aramid	1.44	2700–3600	60–145
Carbon	1.75	3000–6000	200–300
Polypropylene	0.90	27	1.65

**Table 2:** Layout and levels of reinforcement in laminates.

Sample	Stacking Sequence	C (%)	A (%)	Density (kg/m^3^)
AAA	(0°_A_/90°_A_)_7_	0	45	940
CCC	(0°_C_/90°_C_)_7_	55	0	1042
CAC	(0°_C_/90°_C_)_2_/(0°_A_/90°_A_)_3_/(0°_C_/90°_C_)_2_	28	18	1030
ACA	(0°_A_/90°_A_)_2_/(0°_C_/90°_C_)_3_/(0°_A_/90°_A_)_2_	20	24	966

**Table 3:** Comparison of QSPT test results.

Sample	SPR = 2					SPR = 5				
	Hc (mm)	Pmax (kN)	PSS (MPa)	Ea (J)	SEA (J/g)	Hc (mm)	Pmax (kN)	PSS (MPa)	Ea (J)	SEA (J/g)
AAA	5.74	7.75	47.76	48.19	0.89	5.74	8.15	50.26	64.86	1.20
CCC	4.87	4.99	36.29	27.75	0.55	4.87	5.05	36.70	35.79	0.70
CAC	5.61	7.16	45.19	39.18	0.68	5.61	6.53	41.16	46.59	0.81
ACA	6.2	5.89	33.64	38.99	0.65	6.2	5.56	31.72	41.95	0.70

**Table 4:** Laminate samples after a quasi-static penetration attempt performed for two coefficients: SPR = 2 and SPR = 5.

Sample	Front	Back	Cross-Sectional Views of the Samples after the Penetration Tests
	**SPR = 2**		
**AAA**	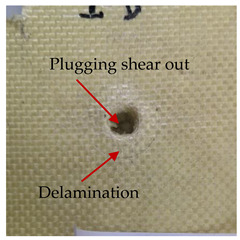	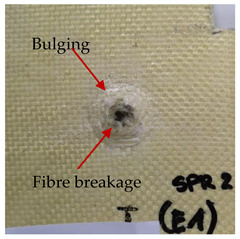	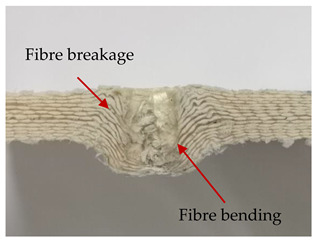
**CCC**	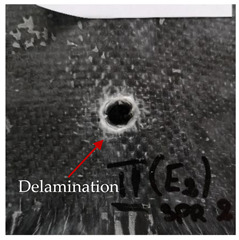	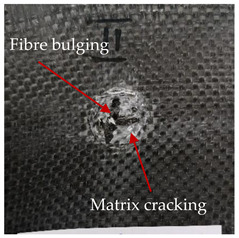	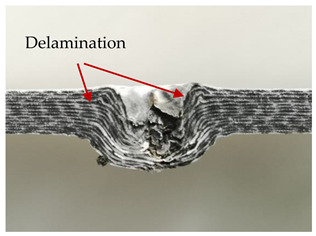
**CAC**	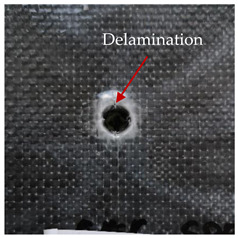	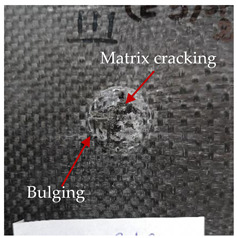	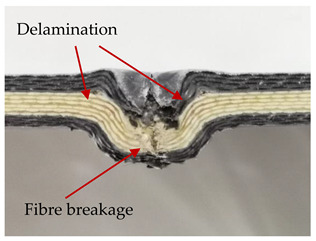
**ACA**	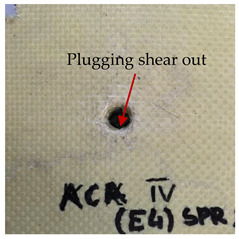	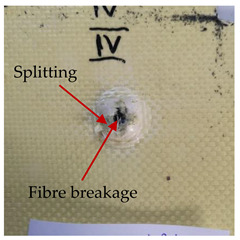	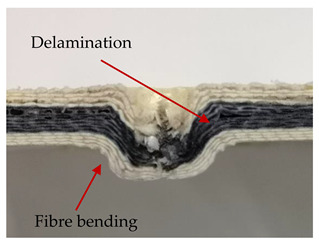
	**SPR = 5**		
**AAA**	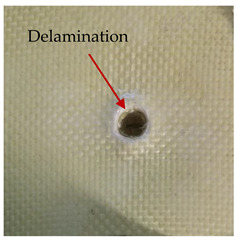	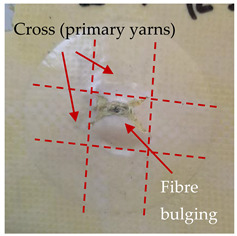	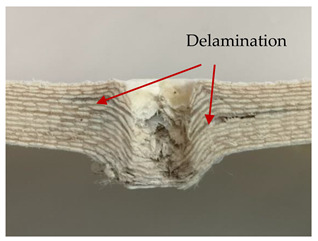
**CCC**	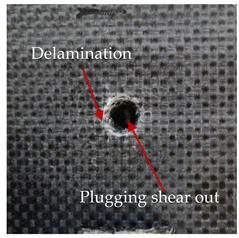	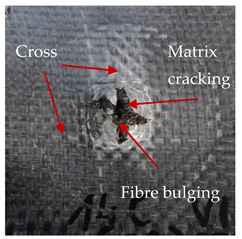	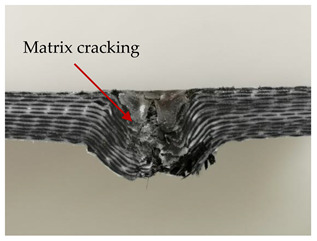
**CAC**	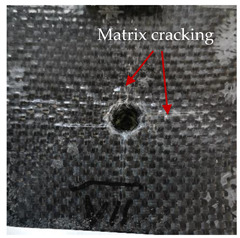	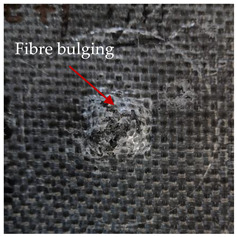	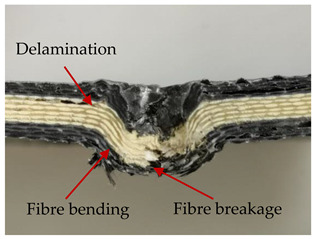
**ACA**	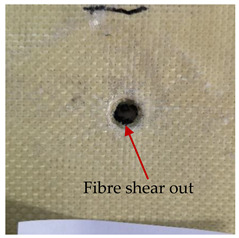	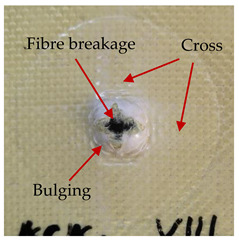	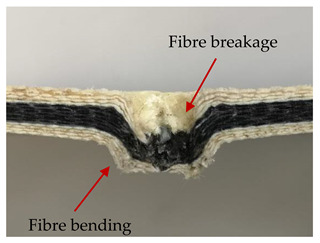

## Data Availability

The data presented in this study are available on request from the corresponding author.
